# Apple endophytic microbiota of different rootstock/scion combinations suggests a genotype-specific influence

**DOI:** 10.1186/s40168-018-0403-x

**Published:** 2018-01-27

**Authors:** Jia Liu, Ahmed Abdelfattah, John Norelli, Erik Burchard, Leonardo Schena, Samir Droby, Michael Wisniewski

**Affiliations:** 1 Chongqing Key Laboratory of Economic Plant Biotechnology, Collaborative Innovation Center of Special Plant Industry in Chongqing, Institute of Special Plants/College of Forestry and Life Science, Chongqing University of Arts and Sciences, Yongchuan, Chongqing, 402160 China; 2 Dipartimento di Agraria, Università Mediterranea di Reggio Calabria, Località Feo di Vito, 89122 Reggio Calabria, Italy; 3 US Department of Agriculture, Agricultural Research Service (USDA-ARS), Kearneysville, WV 25430 USA; 4 Agricultural Research Organization (ARO), the Volcani Center, 50250 Bet Dagan, Israel

**Keywords:** Apple, Endophytic microbiota, Holobiont, Rootstock, Scion cultivar

## Abstract

**Background:**

High-throughput amplicon sequencing spanning conserved portions of microbial genomes (16s rRNA and ITS) was used in the present study to describe the endophytic microbiota associated with three apple varieties, “Royal Gala,” “Golden Delicious,” and “Honey Crisp,” and two rootstocks, M.9 and M.M.111. The objectives were to (1) determine if the microbiota differs in different rootstocks and apple varieties and (2) determine if specific rootstock-scion combinations influence the microbiota composition of either component.

**Results:**

Results indicated that *Ascomycota* (47.8%), *Zygomycota* (31.1%), and *Basidiomycota* (11.6%) were the dominant fungal phyla across all samples. The majority of bacterial sequences were assigned to *Proteobacteria* (58.4%), *Firmicutes* (23.8%), *Actinobacteria* (7.7%), *Bacteroidetes* (2%), and *Fusobacteria* (0.4%). Rootstocks appeared to influence the microbiota of associated grafted scion, but the effect was not statistically significant. Pedigree also had an impact on the composition of the endophytic microbiota, where closely-related cultivars had a microbial community that was more similar to each other than it was to a scion cultivar that was more distantly-related by pedigree. The more vigorous rootstock (M.M.111) was observed to possess a greater number of growth-promoting bacterial taxa, relative to the dwarfing rootstock (M.9).

**Conclusions:**

The mechanism by which an apple genotype, either rootstock or scion, has a determinant effect on the composition of a microbial community is not known. The similarity of the microbiota in samples with a similar pedigree suggests the possibility of some level of co-evolution or selection as proposed by the “holobiont” concept in which metaorganisms have co-evolved. Clearly, however, the present information is only suggestive, and a more comprehensive analysis is needed.

**Electronic supplementary material:**

The online version of this article (10.1186/s40168-018-0403-x) contains supplementary material, which is available to authorized users.

## Background

Apple (*Malus* x *domestica*) is considered one of the most important fruit crops in the world with an annual production exceeding 80 million tons (http://faostat.fao.org). Commercial production typically utilizes the use of a selected scion cultivar grafted on a specific rootstock. This horticultural practice has a wide variety of benefits in regard to size control and related management practices, disease resistance, and fruit quality traits (taste, size, firmness, storability, etc.). Malling 9 (M.9) is a commonly used rootstock. Although it has a good size control, which results in tree size of 45–50% of a standard tree size, it is susceptible to fire blight and wooly apple aphid, but fairly resistant to crown and other root rots. In contrast, Malling Merton 111 (M.M.111) rootstock has resistance to wooly apple aphid and is moderately resistant (tolerant) to fire blight, as well as crown and root rot. Nevertheless, it does not exert much size control, being classified as a semi-dwarf, with resulting grafted trees being about 85 to 100% the size of ungrafted trees. The various attributes that contribute to disease resistance and size control, however, have not always been clearly elucidated, though specific genetic markers for various important traits in apple have been established [1–4].

High-throughput amplicon sequencing has proven to be an excellent tool in studying the microbial diversity of several environments, including the soil [5], phyllosphere [6, 7], carposphere [8], and rhizosphere [9]. In regard to apples, the most detailed studies have been conducted in relation to the rhizosphere and plant pathogens associated with apple replant disease [10–12]. The microbiota of apple flower and fruit has also been characterized. Shade et al. [13] reported the succession of changes in microbial community structure over the life of apple flowers and suggested that this information could be used as a basis for developing ecological approaches to disease management. Abdelfattah et al. [8] noted that different portions of an apple fruit (stem-end, peel, wound, and calyx-end), surveyed at the point-of-purchase (supermarket), possess a distinct microbiota and noted a predominance of yeast often associated with dermal disorders (rashes) in humans.

The relationship between the resident microbiota and their plant hosts is essential to understanding agricultural production systems. Bacterial endophyte communities have been shown to be impacted by management practices [14], and the soil microbiome has been reported to influence grapevine-associated microbiota [15, 16]. The stability and uniformity of the composition, as well as the determinants of plant-associated microbiota, will not be clarified in agricultural crops, however, until many additional studies are conducted. In the present study, we describe the endophytic microbiota associated with three apple varieties, “Royal Gala,” “Golden Delicious,” and “Honey Crisp,” and two rootstocks, “M.9” (dwarfing) and “M.M.111” (semi-dwarfing). The objectives of the study were to (1) determine if the microbiota differs in different rootstocks and apple varieties and (2) determine if specific rootstock-scion combinations influence the composition of the microbiota of either component. The compositions of both the fungal and bacterial communities were assessed.

## Results

### High-throughput amplicon sequencing

**Fig. 1:**
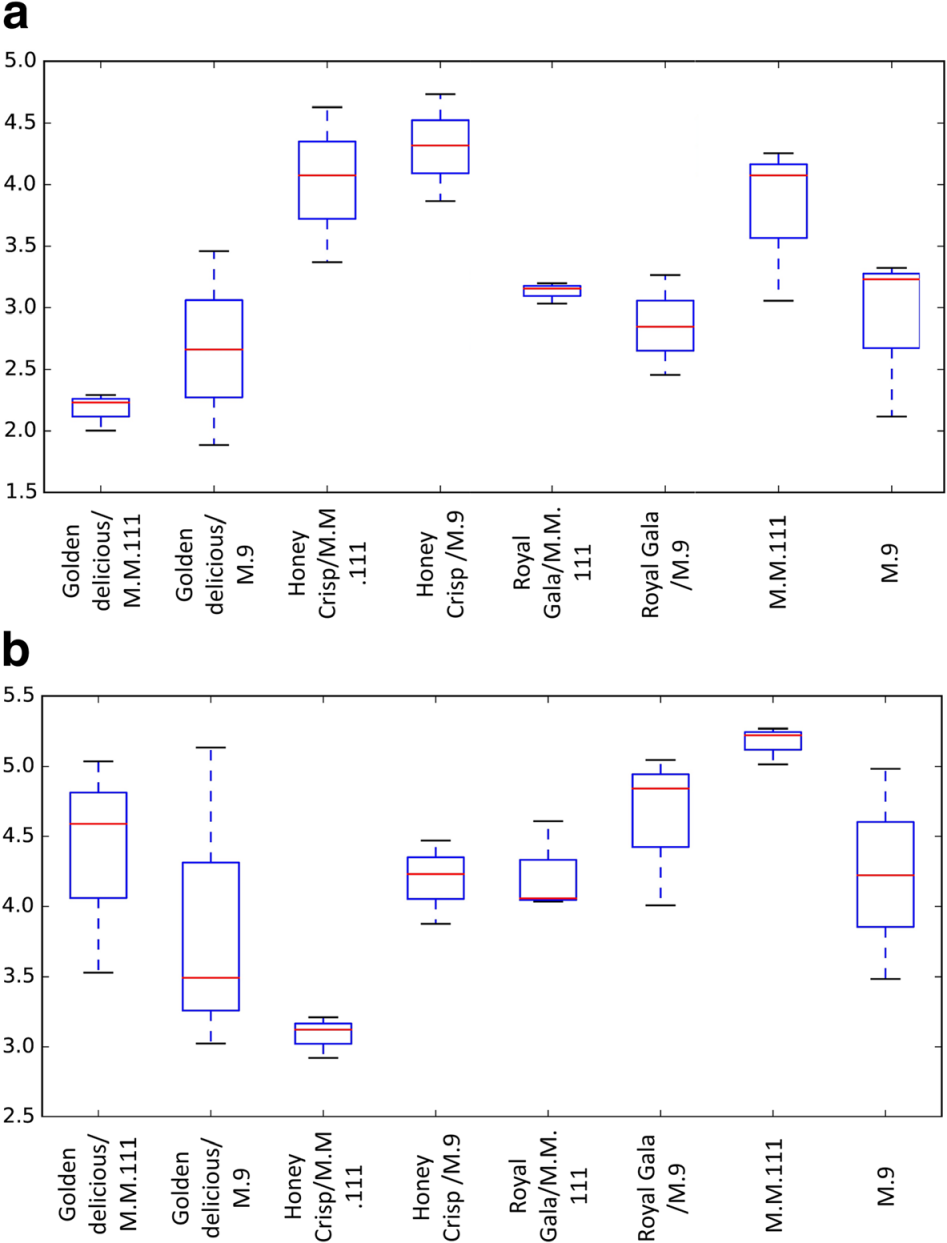
Boxplot illustrating the differences in Shannon diversity measures of the fungal (**a**) and bacterial (**b**) communities in the tested apple cultivars and rootstocks

After paired-end alignments, quality filtering and deletion of chimeric, singletons, and mitochondrial and chloroplast sequences, a total of 156,412 fungal internal transcribed spacer (ITS) and 17,270 bacterial 16S reads were recovered and assigned to 1246 fungal and 513 bacterial operational taxonomic units (OTUs), respectively (Additional file 1: Table S1). It should be noted that the number of identified OTUs reflects the endophytic community and not the entire host microbiome. Based on alpha diversity metrics (Fig. 1), the analysis of the rarefied OTU table to an even sequencing depth indicated that a higher number of fungal OTUs occurred in “Honey Crisp” followed by “Roya Gala.” “Golden Delicious” had the lowest number of fungal OTUs among the three scion cultivars, while the “M.M.111” rootstock had a higher number of fungal OTUs than “M.9” (Fig. 1a). The analysis of the bacterial OTU data also indicated a large degree of variability with the “Honey Crisp”/“M.M.111” samples exhibiting the lowest number of OTUs and the “Royal Gala”/“M.9” samples exhibiting the highest number of OTUs (Fig. 1b).

### Endophytic microbiota of shoots of apple rootstocks and scions

Members of the *Ascomycota* were the dominant fungal phylum across all samples, accounting for 47.8% of the total number of detected sequences (Additional file 2: Figure S1). This was followed by *Zygomycota* (31.1%), *Basidiomycota* (11.6%), *Glomeromycota* (3.3%), and unidentified fungi (5.8%). *Rozellomycota* and *Chytridiomycota* were also detected but at a very low frequency (0.1 and 0.3%, respectively). *Ascomycota* were largely identified as members of the classes *Dothideomycetes* (36.6%), *Sordariomycetes* (5%), and *Eurotiomycetes* (3.3%). The *Zygomycota* was almost exclusively composed of members of the order *Entomophthorales* of the class *Incertae sedis* (31.1%). The *Basidiomycota* were principally represented by members of the *Agaricomycetes* (3.7%) and *Tremellomycetes* (4.1%).

Bacterial OTUs were assigned to *Proteobacteria* (58.4%), *Firmicutes* (23.8%), *Actinobacteria* (7.7%), *Bacteroidetes* (2%), and *Fusobacteria* (0.4%). The *Proteobacteria* were mainly represented by *Gammaproteobacteria* (37.2%), *Betaproteobacteria* (13.3%), and *Alphaproteobacteria* (7.8%), and *Firmicutes* were represented only by the classes *Bacilli* (22.4%) and *Clostridia* (1.4%). *Actinobacteria* (7.7%) was the only bacterial class detected within the phylum *Actinobacteria* (7.7%). Whereas, *Bacteroidetes* (2%) was represented by *Flavobacteriia* (1.1%), *Saprospirae* (0.4%), *Bacteroidia* (0.2%), and *Cytophagia* (0.2%) (Additional file 2: Figure S1).

**Fig. 2:**
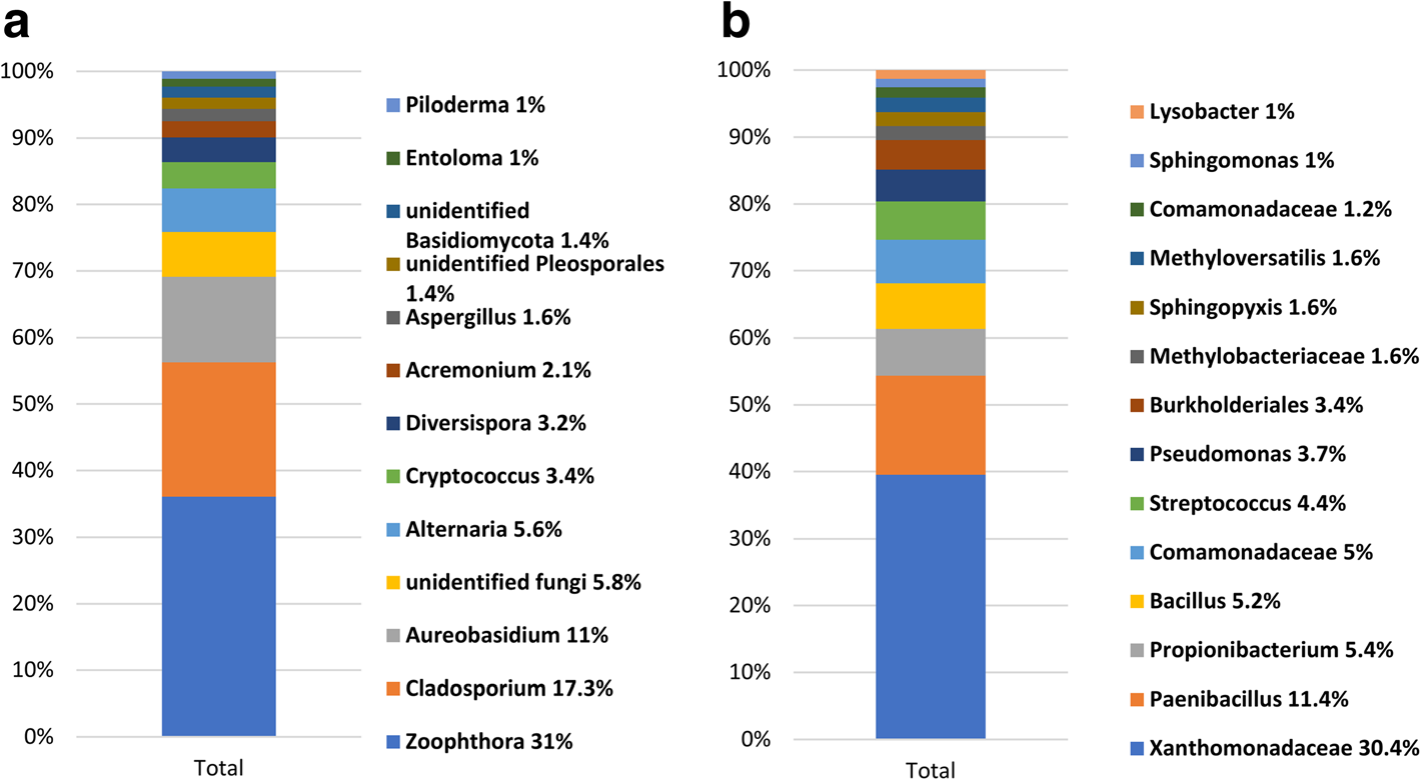
Bar charts showing the relative abundance of most abundant (> 1%) fungal (**a**) and bacterial (**b**) genera detected across all samples

The relative abundance of fungal and bacterial genera detected across all samples was shown in Fig. 2. Overall, the fungal genera *Zoophthora* (31%), *Cladosporium* (17.3%), and *Aureobasidium* (11%) represented more than 59% of the total fungi detected. These genera were followed by *Alternaria* (5.6%), *Cryptococcus* (3.4%), *Diversispora* (3.2%), *Acremonium* (2.1%), and *Aspergillus* (1.6%) (2A). Regarding the bacterial taxa, an unidentified genus from the family *Xanthomonadaceae* (30.4%), *Paenibacillus* (11.4%), *Propionibacterium* (5.4%), and *Bacillus* (5.2%) represented more than 50% of the total bacteria detected. These were followed by genera or unidentified genera that belong to the *Comamonadaceae* (5%), *Streptococcus* (4.4%), *Pseudomonas* (3.7%), *Burkholderiales* (3.4%), *Methylobacteriaceae* (1.6%), *Sphingopyxi*s (1.6%), *Methyloversatilis* (1.6%), *Comamonadaceae* (1.2%), *Sphingomonas* (1%), and *Lysobacter* (1%) (Fig. 2b).

### Differences in the composition of the microbiota in “M.9” vs. “M.M.111” rootstocks

**Fig. 3:**
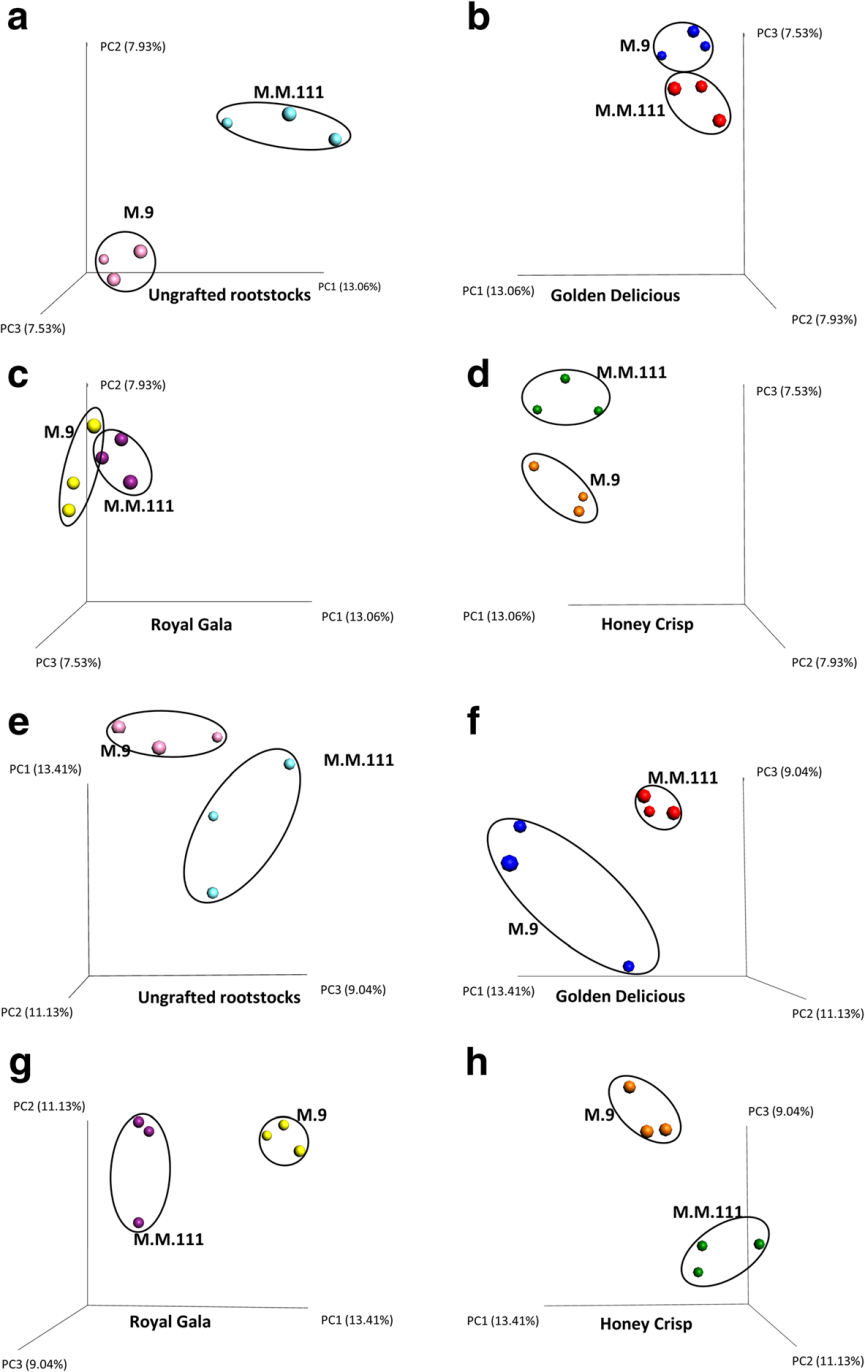
Principle Coordinate Analysis (PCoA) based on Bray Curtis dissimilarity metrics, showing the distance in the fungal (**a**–**d**) and bacterial (**e**–**h**) communities between the same cultivars when grafted on different rootstocks, i.e., M.M.111 or M.9. The dissimilarity metrics are also presented for the “M.9” and “M.M. 111” ungrafted rootstocks (**a**, **e**)

**Fig. 4:**
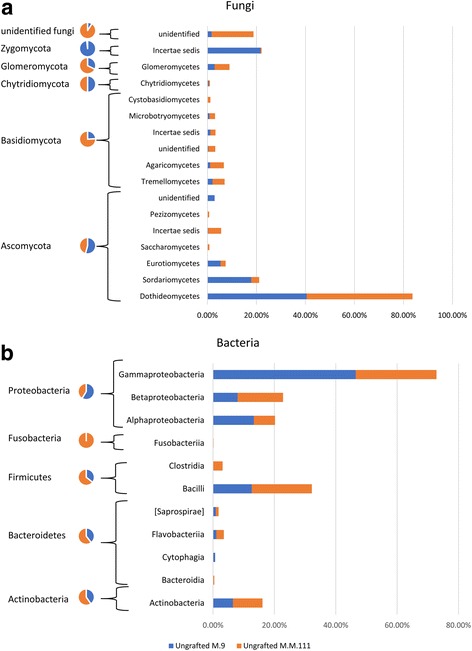
Stacked bar charts showing the relative abundance of most dominant fungal (**a**) and bacterial (**b**) phyla and classes and their distribution between M.9 and M.M.111 rootstocks using a cutoff of 0.1% across all samples

The number of observed fungal OTUs, based on the rarefied OUT table, in “M.9” samples, ranged from 13 to 38 with a mean of 29, while the range in “M.M.111” was 34–57 with a mean of 45. The number of bacterial OTUs in M.9 ranged between 40.5 and 66.5 and a mean of 54.53 and between 60.5 and 67.2 OTUs in M.M.111 with a mean of 63.26 OTUs (Additional file 1: Table S1). The calculated differences in the number of observed species and the Shannon index for both the fungal and bacterial diversity in ungrafted “M.9” vs. ungrafted “M.M.111” were not statistically significant (*P* > 0.05). Similarly, beta diversity, based on Bray Curtis index, indicated that the two rootstocks did not differ significantly in their bacterial or their fungal communities (*P* > 0.05). Nevertheless, when the beta diversity distance matrix was subjected to Principle Coordinate Analysis PCoA, the fungal and bacterial OTUs from the two rootstocks clearly clustered into different groups, suggesting that the sample size or read depth was not sufficient enough to reveal the differences statistically (Fig. 3a, e). Despite the lack of statistical significance in beta diversity, the results of Kruskal-Wallis comparisons showed that “M.9,” at the phylum level, had a significantly higher relative abundance (RA) of *Ascomycota* and *Zygomycota* (Additional file 3: Table S2). In contrast, M.M.111 had a higher RA of *Glomeromycota* and *Basidiomycota*, than “M.9,” as well as a much higher RA of unidentified fungi (Fig. 4a). Regarding the bacterial phyla, M.M.111 had a higher RA of *Actinobacteria*, *Bacteroidetes*, and *Firmicutes*, than “M.9,” and a much higher RA of *Fusobacteria*. Whereas, “M.9” had higher RA of *Proteobacteria* than “M.M.111” (Fig. 4b). Fungal genera, such as *Zoophthora*, *Malassezia*, *Phaeococcomyces*, *Jattaea*, *Entoloma*, and unidentified groups of *Sordariomycetes* and *Pyronemataceae* as well as the bacterial genera of *Bacillus*, *Streptococcus*, and unidentified groups of *Planococcaceae*, *Methylobacteriaceae*, *Burkholderiales*, and *Comamonadaceae*, were detected at significantly different levels of RA between the two rootstocks (Additional file 3: Table S2).

### Comparisons between scion cultivars

The comparison between the three scions using Student’s *t* test revealed that there were significant differences in the alpha diversity (based on the Shannon index) of the endophytic OTUs present in “Golden Delicious,” “Honey Crisp,” and “Royal Gala” (Additional file 4: Table S3). No significant differences were observed, however, between “Royal Gala” and “Golden Delicious,” except in the number of observed OTUs. The beta diversity of the endophytic fungal microbiota of “Royal Gala” vs. “Honey Crisp,” and “Golden Delicious” vs. “Honey Crisp” was also significantly different. No statistically significant differences were observed, however, between the cultivars in regard to the alpha or beta diversity of the bacterial OTUs (Additional file 4: Table S3).

**Fig. 5:**
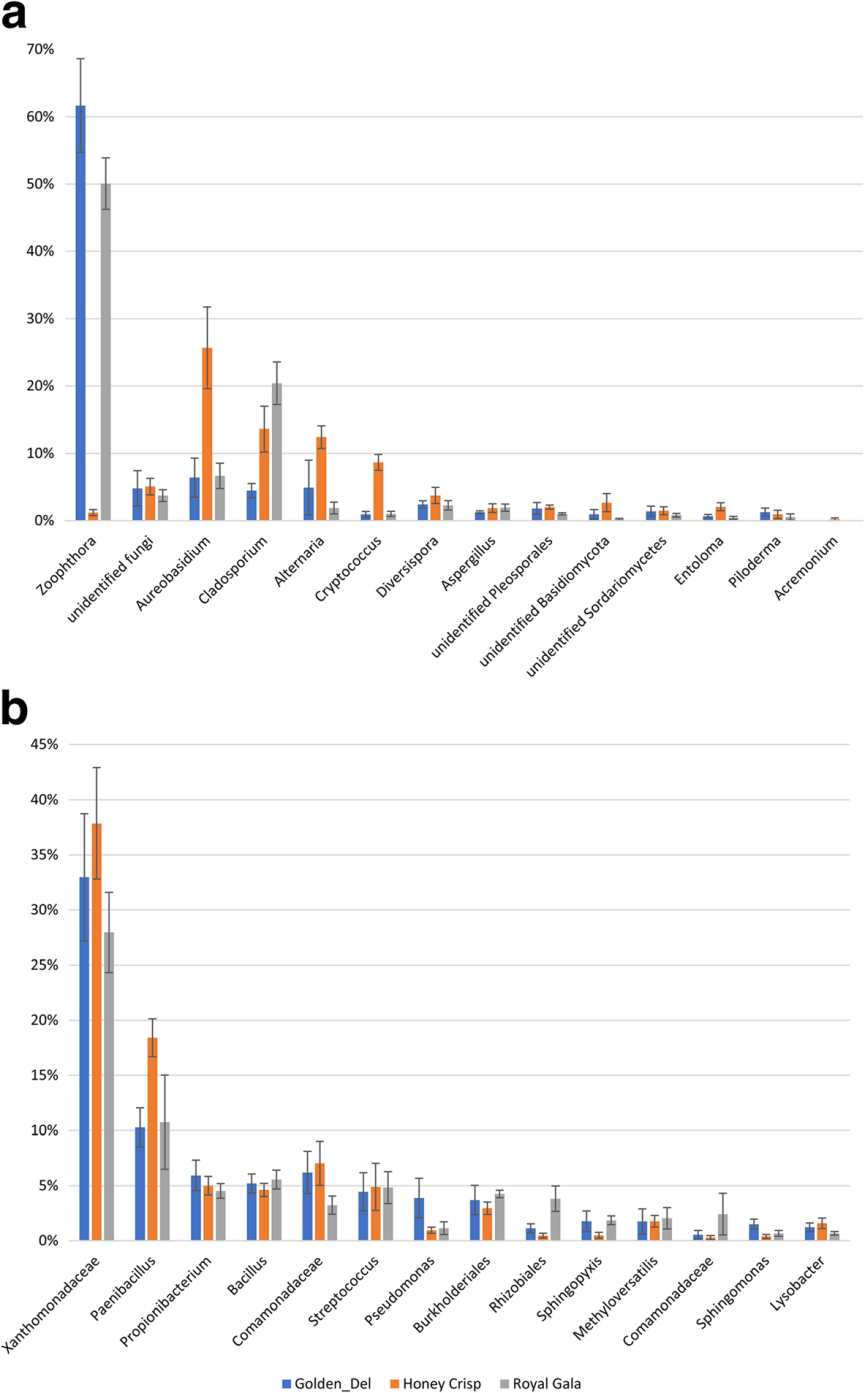
A comparison of the relative abundance of the most abundant fungal (**a**) and bacterial (**b**) taxa (≥ 1%) in the three different scion cultivars, “Golden Delicious,” “Royal Gala,” and “Honey Crisp”

The composition and the relative abundance of the individual fungal OTUs were very similar in “Royal Gala” and “Golden Delicious,” sharing most of the same taxa with comparable RA (Fig. 5a). The composition and RA of the fungal OTUs in “Honey Crisp,” however, was significantly different from the other two cultivars. For instance, both “Royal Gala” and “Golden Delicious” had a very high RA of *Zoophthora* 50.10 and 61.70%, respectively, compared to only 1.20% in “Honey Crisp.” In contrast, “Honey Crisp” had higher RA of *Aureobasidium*, *Alternaria*, and *Cryptococcus* compared to “Royal Gala” and “Golden Delicious” (Fig. 5a). No significant differences were observed in the bacterial composition between the different cultivars (Fig. 5b).

### Impact of different rootstocks on the microbiota of scion varieties

**Fig. 6:**
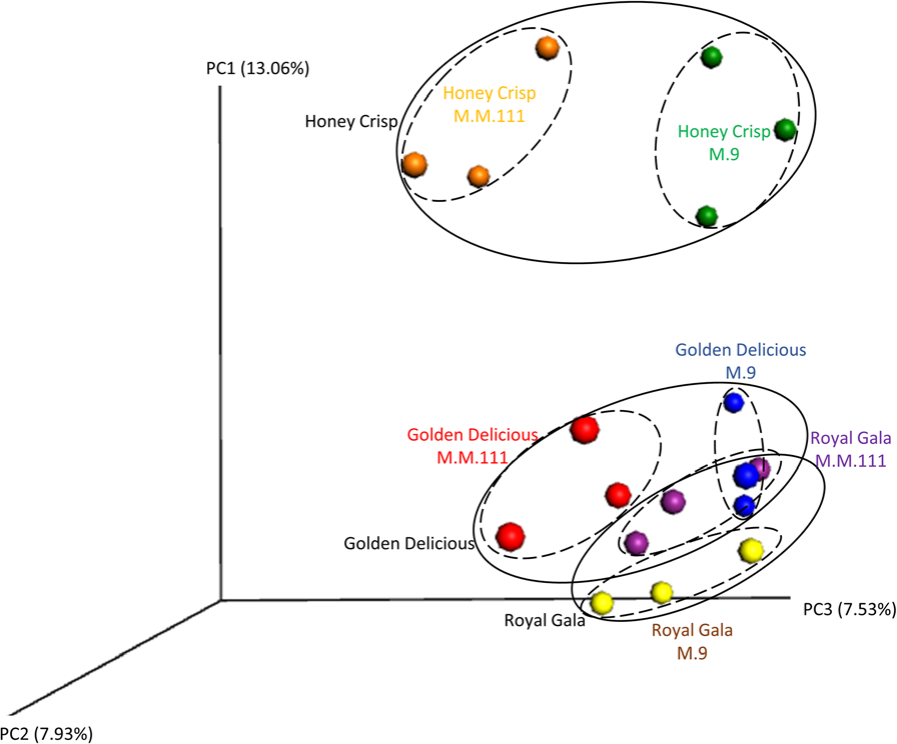
Principle Coordinate Analysis (PCoA) based on Bray Curtis dissimilarity metrics, showing the distance in the fungal communities of all three scion cultivars (“Golden Delicious,” “Royal Gala,” and “Honey Crisp”) grafted on “M.9” or “M.M.111” rootstocks. Note that cultivars with a more closely related pedigree (“Golden Delicious” and “Royal Gala”) cluster closer to each other than the scion cultivar (“Honey Crisp”) with a less-related pedigree

Grafting the different varieties on the two different rootstocks did not significantly influence the alpha or beta diversity of the fungal or bacterial microbiota in any of the scions (Additional file 4: Table S3). However, when the samples were plotted on a PCoA using Bray Curtis data, they distinctly clustered based on both the scion (Fig. 6) and the rootstock (Fig. 3). ANOVA analysis of the PCoA data also indicated significant differences between the beta diversity of fungal OTUs between cultivars on different rootstocks (Additional file 5: Table S4). For example, the beta diversity of fungal OTUs was significantly different between “Golden Delicious”/“M.9” vs. “Honey Crisp”/“M.9,” ungrafted “M.M.111,” and “Honey Crisp”/“M.M.111.” In contrast to the alpha and beta diversity results, according to Kruskal-Wallis comparisons, several fungal and bacterial genera also exhibited significantly different levels of RA within each of the three cultivars when grafted on different rootstocks (Additional file 6: Tables S5–7).

## Discussion

**Fig. 7:**
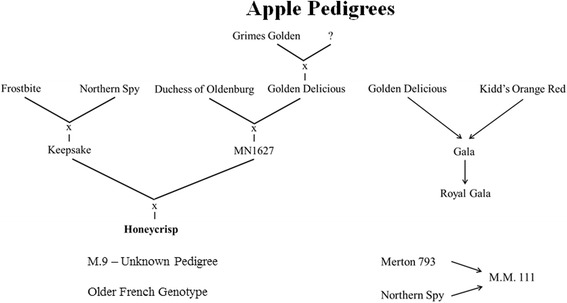
Pedigree of “Honey Crisp” and “Royal Gala” scion cultivars and “M.9” and “M.M.111” rootstocks. Note that “Royal Gala” is more closely related to “Golden Delicious” than “Honey Crisp.” The two rootstocks also appear to have little in common in their pedigree, although the exact pedigree of “M.9” has not been determined

In the present study, we were interested in determining if different scions and rootstocks exhibited different compositions in their endophytic microbial communities (fungi and bacteria) and if different rootstocks could influence the composition of the microbiome of a grafted scion cultivar. The results indicate that genotype (rootstock and scion) does have an influence on the composition of the fungal endophytic community but not the bacterial community. PCoA plots (Figs. 3 and 6) revealed that the fungal OTUs of different rootstocks cluster into distinct groups. Rootstock genotype, however, did not appear to significantly influence the composition of the fungal endophytic community within a cultivar. The lack of distinct, statistically significant, alpha and beta diversity due to rootstock genotype was most likely due to the sample size used in the study, which was limited by the availability of the experimental material. Nevertheless, the influence of genotype (rootstock and scion) is very evident. Interestingly, the composition of the fungal microbiota of “Golden Delicious” and “Royal Gala” could not be separated from each other (Additional file 4: Table S3; Fig. 6). Both varieties, however, were distinctly separated from “Honey Crisp,” regardless of the genotype of the rootstock. In this regard, “Royal Gala” and “Golden Delicious” have a distinctly different pedigree from “Honey Crisp” (Fig. 7), suggesting that genotype in apple does influence the composition of the associated microbiome. Notably, the pedigrees of the two rootstocks examined in the present study also appear to be unrelated, although little is known about the pedigree of “M.9.” No such distinctions could be made for the endophytic bacterial community. Based on the findings of the current study, a more comprehensive study of the relationship between apple pedigree and the endophytic fungal microbiome is in progress, where the endophytic microbiota from several apple progenitor species and apple evolutionary clusters (based on comparisons of their chloroplast genome) are being characterized.

Other studies have reported mixed results in regard to the impact of genotype on the composition of the associated microbiota. Weinert et al. [17] reported that tuber-associated bacteria were only weakly affected by genotype. Leff et al. [18] in a study of 33 strains of sunflower (*Helianthus annus*) that differed in their degree of domestication found that plant-associated fungal communities in the rhizosphere were more strongly influenced by host genetic factors and plant breeding than bacterial communities. They also found that there was a minimal vertical transmission of fungi from seeds to adult plants. The findings of the present study are in agreement with the results of the latter study. Bálint et al. [19] reported that host genotype had a structuring effect on the composition of foliar fungal communities in balsam poplar (*Populus balsamifera*) trees planted in a common garden. They hypothesized that there was either a filtering mechanism that allowed trees to be selective in their recruitment of fungi from the environment or that the fungal organisms were present systemically, thus allowing them to be “passed on” during successive clonal propagations. Sun et al. [20] found that host genotype (species) explained 30.1% of the variability (as determined by redundancy analysis) in the endophytic composition of three forest species (*Betula platyphylla*, *Quercus liaotungensis*, and *Ulmus macrocarpa*) in a mixed temperate forest. Pérez-Izquierdo et al. [21] reported that tree genotype was pivotal in structuring fungal communities in Mediterranean pine forests. Collectively, these studies suggest a degree of co-evolution or co-inheritance between a genotype and its associated microbiota (holobiont concept); however, more detailed and comprehensive studies will need to be conducted in apple to support such an inference.

This is the first examination of the endophytic population of apple using high-throughput amplicon sequencing. Beneficial symbionts, such as *Diversispora* and *Piloderma*, are recognized as endophytes that have a beneficial influence on the growth and stress tolerance of plants [22, 23]. These two genera were more abundant in the “M.M.111” rootstock than in the “M.9,” where their presence was low or absent. It would be interesting to determine if the growth promoting taxa present in “M.M.111” and their absence in “M.9” plays a role in the growth-related attributes associated with these two different rootstocks. M.9 had a higher RA of genera such as *Zoophthora*, *Acremonium*, and species from the order *Eurotiales* that are either saprophytic or antagonists of insects. The high RA of *Zoophthora* was reflected in the dominant presence of *Zygomycota* in “Royal Gala” and “Golden Delicious.” While the dominant presence of the *Zygomycota* in these two cultivars contradicts the general belief that *Ascomycota* is the most dominant, plant-associated phylum, *Ascomycota*, was still the predominant phylum in the combined samples collectively.

The consistent high prevalence of *Xanthomonadaceae* in all rootstocks and scions was surprising. This family contains a long list of phytopathogenic bacteria including *Xanthomonas* species and *Xylella fastidiosa* which causes disease in a wide range of important agricultural crops*. Paenibacillus* has been detected in various environments but is commonly recognized as a predominantly endophytic bacterium in woody plants [24]. It also functions in growth promotion and nitrogen fixation in plants [25]. Interestingly, like the growth beneficial fungi, this growth-promoting bacterium was generally found to be more abundant in “M.M.111,” the vigorous rootstock. Members of the *Propionibacterium* had a similar abundance pattern as *Paenibacillus. Propionibacteria* are used in the production of vitamin B12 and vitamin B2 and the production of porphyrinogen and tetrapyrrole compounds and exopolysaccharides [26]. As the name indicates, these bacteria are known to synthesize propionic acid, which can solubilize phosphate. Since more than 90% of phosphate present in the soil or absorbed by the plant is in an insoluble form, these bacterial taxa may play a role in making phosphorus available to the plant. *Methylobacterium* species are ubiquitous in the natural environment, both as free-living organisms in soil and water, but also on the phylloplane and in leaf, stem, and root tissues of plants. Some induce plant leaf and root nodule formation and can promote plant growth by the production of auxins [27]. Some species of *Burkholderiales* are associated with plants and are known to have growth-promoting effects [28]. *Burkholderia* species are common rhizosphere constituents of major agricultural crops including maize, rice, sugarcane, wheat, tomato, and potato. A valuable feature of some growth-promoting *Burkholderia* species is their capacity for biological nitrogen fixation, siderophore production, inorganic phosphates solubilization, indoleacetic acid production, and phytopathogen inhibition [29].

## Conclusions

A greater diversity of fungi than bacteria was identified in the analysis of the microbiota of various apple rootstock/scion combinations. In regard to the fungi, a distinct influence of genotype (both rootstock and scion) on the composition of the fungal microbiota was observed as well as in the relative abundance of specific genera or species. The compositions of the fungal taxa in “Golden Delicious” and “Royal Gala,” two scions closely-related by pedigree, were determined to be more similar to each other than they were to the composition of the fungal taxa in “Honey Crisp,” a scion more distantly-related by pedigree. A similar relationship in bacterial taxa could not be established. Interestingly, “M.M.111” rootstock, which does not have strong growth control properties, had a great number of beneficial and growth promoting fungal and bacterial taxa than “M.9” rootstock which does exhibit significant size control. Also of interest was the presence of high relative abundance of the genus *Zoophthora* in “Golden Delicious” and “Royal Gala,” a fungal genus generally recognized as an insect pathogen. Evidence for a genotype-specific impact on the composition of the endophytic microbiome in apple shoots suggests that further studies should be conducted to determine if the “holobiont” concept is supported in apple. Only a few microbiome-related studies have been conducted on temperate fruit trees, and it is expected that future research will have important implications on many aspects of tree physiology and cultivar development.

## Methods

### Experimental design

High-throughput amplicon sequencing of the endophytic microbiota was conducted on two-year-old, clonally propagated and grafted apple trees grown in pots in a greenhouse located at the US Department of Agriculture-Agricultural Research Service (USDA-ARS), Appalachian Fruit Research Station, Kearneysville, WV, USA. The trees were part of a longer-term study on tree growth and root architecture and were maintained at ambient light conditions and temperatures, watered, and fertilized on a regular basis. Trees of self-rooted “M.9” and “M.M.111” rootstocks and three apple scion varieties (“Honey Crisp,” “Golden Delicious,” and “Royal Gala”) grafted on either rootstock were used in the study. The microbial analysis was conducted on a total of 24 stem samples (three varieties on two rootstocks and two rootstocks × three biological replicates), destructively sampled, frozen, and ground in liquid nitrogen, prior to DNA extraction.

Several shoots (20 cm in length) were collected from each sampled tree, surface sterilized with 5% sodium hypochlorite (*v*:*v*), and rinsed three times in sterile water. The bark of each shoot was then removed with a sterile razor. The remaining xylem tissues were then immersed in liquid nitrogen and maintained at − 20 °C until lyophilization. Lyophilized samples were homogenized in a 2010 Geno/Grinder (SPEX SamplePrep, Metuchen, NJ, USA) using autoclaved metal beads. Total DNA was extracted from the ground xylem tissues using a Wizard Genomic DNA Purification Kit according to the manufacturer’s protocol (Promega, Madison, WI, USA). The quantity of each of the DNA samples was determined using a spectrophotometer (Nanodrop; Thermo Fisher Scientific Inc.), and the total DNA concentration was adjusted to 5.0 ng μL^−1^. The fungal ITS2 region was amplified using the universal primers ITS3/KYO2 and ITS4 to amplify the ITS2 region of the ribosomal DNA [30]. The bacterial 16S region was amplified using the protocol described by Lundberg et al. [31], including the use of a pair of peptide-nucleic-acids (PNA) that were incorporated into the PCR amplification in order to reduce the generation of non-target chloroplast and mitochondrial amplicons. The universal 16S primer pair 515F and 806R was used to generate bacterial-derived 16S amplicons [31]. All primers were modified to include Illumina adaptors (www.illumina.com). PCR reactions were conducted in a total volume of 25 μL containing 12.5 μL of KAPA HiFi HotStart ReadyMix (Kapa Biosystems, Wilmington, MA, USA), 1.0 μL of each primer (10 μM), 2.5 μL of DNA template, and 8.0 μL nuclease-free water. The reactions were incubated in a T100 thermal cycler (Bio-Rad) for 3 min at 98 °C followed by 30 cycles of 30 s at 95 °C, 30 s at 50 °C, and 30 s at 72 °C. All reactions ended with a final extension of 1 min at 72 °C. Nuclease-free water (QIAGEN, Valencia, CA, USA) replaced template DNA in negative controls. All amplicons and amplification mixtures including negative controls were sequenced on a MiSeq platform using V2 chemistry (Illumina, San Diego, CA, USA).

### Data analysis

Paired-end reads were merged using PEAR 0.9.6 Paired-End reAd mergeR [32] and default parameters. The CLC genomics workbench V8 (Qiagen) was used for primer and quality trimming with a minimum of Q20. Sequences without either primer were discarded. Chimeric sequences were identified and filtered using VSEARCH 1.4.0 [33]. The UCLUST algorithm [34] of the software package QIIME 1.9.1 [35] was used to cluster sequences at a similarity threshold of 97% against UNITE dynamic database [36] released on 31 January 2016 for ITS reads and Greengenes Database [37] for the 16S reads. Sequences that failed to cluster against the database were de novo clustered using the same algorithm. The most abundant sequences in each OTU were selected as representative sequences and used for the taxonomic assignment using the BLAST algorithm [38] as implemented in QIIME 1.9.1.

The OTU table was normalized by rarefaction to an even sequencing depth in order to remove sample heterogeneity. The rarefied OTU table was used to calculate alpha diversity indices including observed species (Sobs), Chao1, and Shannon metrics. MetagenomeSeq’s cumulative sum scaling (CSS) was used as a normalization method for other downstream analyses, including taxa relative abundance, β-diversity, and group significance [39]. Alpha diversities were compared based on a two-sample *t* test using non-parametric (Monte Carlo) methods and 999 Monte Carlo permutations (999). The CSS-normalized OTU table was analyzed using Bray Curtis metrics [40] and utilized to evaluate the β-diversity and construct PCoA plots [41]. Only OTUs that were found in at least 50% of the samples were used to calculate the differential OTU abundance using the *t* test and the Kruskal-Wallis test [42]. In all tests, significance was determined using 999 Monte Carlo permutations, and the false discovery rate (FDR) was used to adjust the calculated *P* value, and when the FDR-*P* ≤ 0.05, it was considered significant. PC1 and PC2 scores of the PCoA results were subjected to ANOVA with the Fisher’s LSD post hoc test to determine the significance of the sample clustering using IBM SPSS Statistics version 22 (IBM Corp., Armonk, NY).

## Additional files

Additional file 1: Table S1. Summary of the investigated samples in the current study. The table includes the number of reads and OTUs observed in each sample as well as Shannon index results of both ITS and 16S data. (DOCX 20 kb)

Additional file 2: Figure S1. Pie charts illustrating the percent relative abundance of different fungal and bacterial phyla and classes across all samples. (TIFF 2290 kb)

Additional file 3: Table S2. The results of Kruskal-Wallis test comparing genera relative abundance of CSS-normalized OTUs table when considering only the core microbiome (taxa present at least in 50% of the samples). (DOCX 16 kb)

Additional file 4: Table S3. *P* values of the comparisons between scions regardless of the rootstock they were grafted on using Alpha (observed OTUs and Shannon index) and beta diversity based on Bray Curtis metric. (DOCX 18 kb)

Additional file 5: Table S4. ANOVA analysis of the PCoA showing the impact of the grafting on the apple cultivars. (DOCX 17 kb)

Additional file 6: The fungal and bacterial genera that were significantly present at different abundance, according to Kruskal-Wallis comparisons. Description - Table S5. The fungal and bacterial genera that were significantly present at different abundance between “Golden Delicious” when grafted on M.M.111 and M.9. Table S6. The fungal and bacterial genera that were significantly present at different abundance between “Royal Gala” when grafted on M.M.111 and M.9. Table S7. The fungal and bacterial genera that were significantly present at different abundance between “Honey Crisp” when grafted on M.M.111 and M.9. (DOCX 18 kb)
