# Supplementary material for: Apple endophytic microbiota of different rootstock/scion combinations suggests a genotype-specific influence
Source: Microbiome. 2018 Jan 27;6:18. doi: 10.1186/s40168-018-0403-x (PMC5787276; doi:10.1186/s40168-018-0403-x)
Supplement: Supplementary file 1 — Summary of the investigated samples in the current study. The table includes the number of reads and OTUs observed in each sample as well as Shannon index results of both ITS and 16S data. (DOCX 20 kb) [file 40168_2018_403_MOESM1_ESM.docx]

**Table S1.** Summary of the investigated samples in the current study. The table includes the number of reads and OTUs observed in each sample as well as Shannon index results of both ITS and 16S data.

| Description | Sample ID | No. ITS Seq. | No. 16S Seq. | Sobs^a^  ITS | Sobs^a^  16S | Sobs^a*^  ITS | Sobs^a*^  16S | Shannon^*^  ITS | Shannon^*^ 16S |
| --- | --- | --- | --- | --- | --- | --- | --- | --- | --- |
| **‘Royal Gala’/‘M.9’** | S1 | 4946 | 265 | 177 | 78 | 36 | 62.6 | 2.85 | 5.05 |
| **‘Royal Gala’/‘M.9’** | S2 | 6438 | 808 | 228 | 129 | 38.4 | 52.7 | 3.26 | 4.01 |
| **‘Royal Gala’/‘M.9’** | S3 | 2801 | 340 | 134 | 82 | 34.7 | 58.5 | 2.46 | 4.84 |
| **Ungrafted/‘M.9’** | S4 | 84690 | 1554 | 353 | 120 | 13 | 40.5 | 2.12 | 3.48 |
| **Ungrafted/‘M.9’** | S5 | 1708 | 182 | 119 | 67 | 38 | 66.5 | 3.23 | 4.98 |
| **Ungrafted/‘M.9’** | S6 | 4093 | 395 | 155 | 91 | 34.7 | 56.6 | 3.32 | 4.22 |
| **‘Golden Del.’/‘M.9’** | S7 | 1571 | 2986 | 114 | 150 | 42.8 | 33.5 | 3.46 | 3.02 |
| **‘Golden Del.’/‘M.9’** | S8 | 5405 | 455 | 136 | 65 | 29.6 | 38.7 | 1.88 | 3.49 |
| **‘Golden Del.’/‘M.9’** | S9 | 3808 | 408 | 133 | 104 | 27.5 | 66.8 | 2.66 | 5.13 |
| **‘Honey Crisp’/‘M.9’** | S10 | 296 | 317 | 58 | 74 | 54.9 | 52.8 | 4.32 | 4.47 |
| **‘Honey Crisp’/‘M.9’** | S11 | 265 | 426 | 57 | 72 | 55.8 | 48.3 | 4.73 | 4.23 |
| **‘Honey Crisp’/‘M.9’** | S12 | 1008 | 452 | 117 | 81 | 52.1 | 45.6 | 3.87 | 3.88 |
| **‘Royal Gala’/‘M.M.111’** | S13 | 1877 | 394 | 95 | 80 | 35.5 | 47.3 | 3.16 | 4.06 |
| **‘Royal Gala’/‘M.M.111’** | S14 | 4550 | 889 | 162 | 121 | 37.2 | 53.7 | 3.20 | 4.61 |
| **‘Royal Gala’/‘M.M.111’** | S15 | 1849 | 637 | 113 | 97 | 41.4 | 46.4 | 3.03 | 4.03 |
| **Ungrafted/‘M.M.111’** | S16 | 739 | 458 | 78 | 98 | 44.3 | 60.5 | 4.25 | 5.01 |
| **Ungrafted/‘M.M.111’** | S17 | 460 | 238 | 73 | 77 | 52.6 | 67.2 | 4.08 | 5.27 |
| **Ungrafted/‘M.M.111’** | S18 | 2381 | 204 | 123 | 66 | 37.9 | 62.1 | 3.06 | 5.22 |
| **‘Golden Del.’/‘M.M.111’** | S19 | 2741 | 205 | 110 | 67 | 28.6 | 62.3 | 2.23 | 5.04 |
| **‘Golden Del.’/‘M.M.111’** | S20 | 8467 | 374 | 167 | 80 | 28.6 | 50.3 | 2.29 | 4.59 |
| **‘Golden Del.’/‘M.M.111’** | S21 | 9264 | 939 | 217 | 104 | 28.8 | 41.9 | 2.00 | 3.53 |
| **‘Honey Crisp’/‘M.M.111’** | S22 | 880 | 1584 | 120 | 98 | 57 | 31.1 | 4.63 | 2.92 |
| **‘Honey Crisp’/‘M.M.111’** | S23 | 3043 | 2067 | 187 | 115 | 44.7 | 32.6 | 4.07 | 3.12 |
| **‘Honey Crisp’/‘M.M.111’** | S24 | 3132 | 693 | 137 | 78 | 34.4 | 35.9 | 3.37 | 3.21 |

^a^ Sobs = species observed; *Index calculated at an even sequencing depth of 250 sequences/sample in fungi and 180 sequence/sample in bacteria.
