# Supplementary figures and images for: Apple endophytic microbiota of different rootstock/scion combinations suggests a genotype-specific influence
Source: Microbiome. 2018 Jan 27;6:18. doi: 10.1186/s40168-018-0403-x (PMC5787276; doi:10.1186/s40168-018-0403-x)

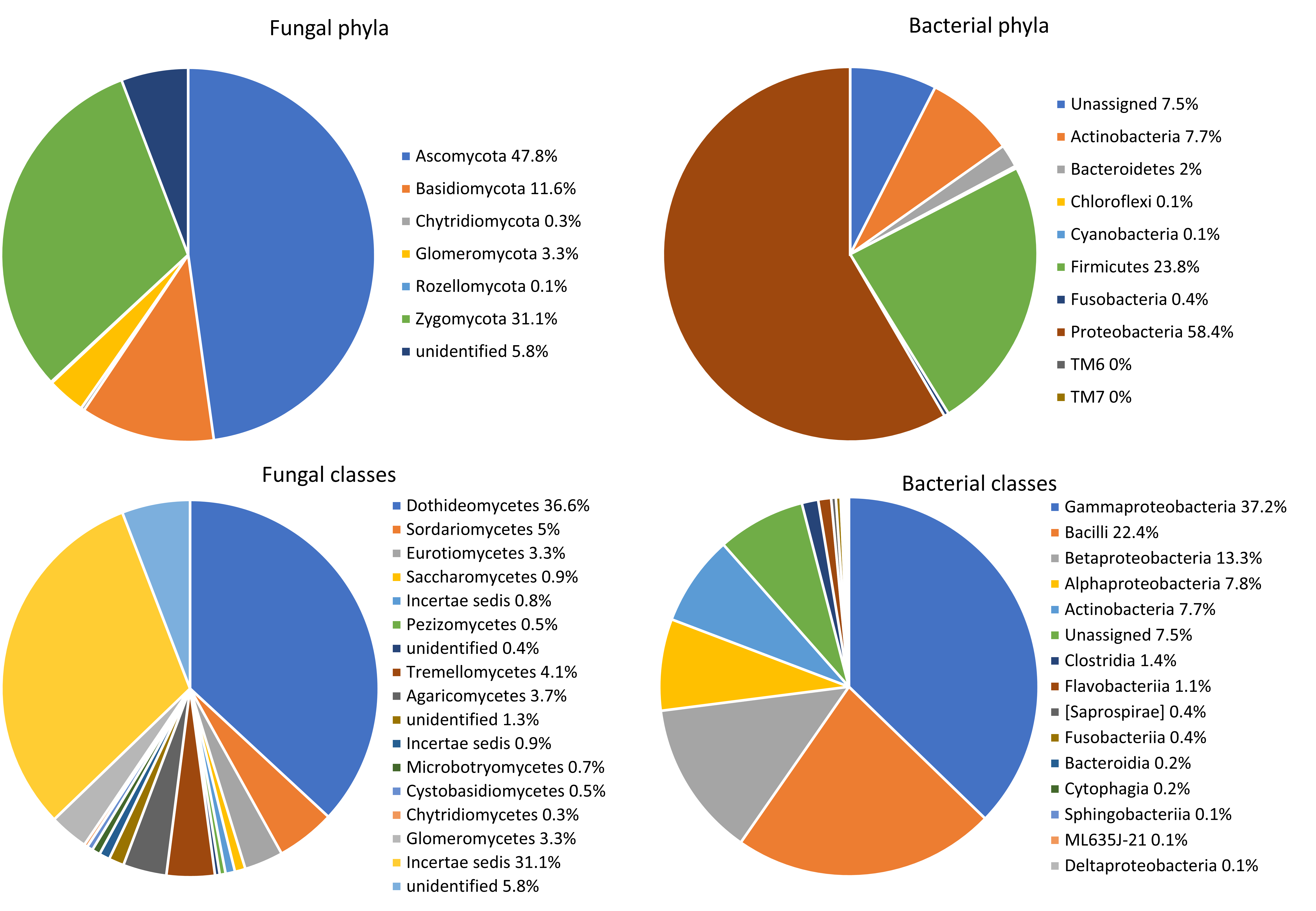

Supplement: Supplementary file 2 — Pie charts illustrating the percent relative abundance of different fungal and bacterial phyla and classes across all samples. (TIFF 2290 kb) [file 40168_2018_403_MOESM2_ESM.tif]
