# Supplementary material for: Apple endophytic microbiota of different rootstock/scion combinations suggests a genotype-specific influence
Source: Microbiome. 2018 Jan 27;6:18. doi: 10.1186/s40168-018-0403-x (PMC5787276; doi:10.1186/s40168-018-0403-x)
Supplement: Supplementary file 3 — The results of Kruskal-Wallis test comparing genera relative abundance of CSS-normalized OTUs table when considering only the core microbiome (taxa present at least in 50% of the samples). (DOCX 16 kb) [file 40168_2018_403_MOESM3_ESM.docx]

**Table S2.**The results of Kruskal Wallis test comparing genera relative abundance, of CSS normalized OTUs table, when considering only the core microbiome (taxa present at least in 50% of the samples).

|  | **OTU** | **Test-Statistic** | ***P*-value** |
| --- | --- | --- | --- |
| **Fungal** | Ascomycota | 3.857 | 0.0495 |
|  | Zygomycota | 3.857 | 0.0495 |
|  | unidentified *Phaeococcomyces* | 4.355 | 0.0369 |
|  | unidentified *Sordariomycetes* | 4.355 | 0.0369 |
|  | unidentified *Pyronemataceae* | 3.857 | 0.0495 |
|  | *Jattaea* | 3.857 | 0.0495 |
|  | *Entoloma* | 3.857 | 0.0495 |
|  | *Malassezia* | 3.857 | 0.0495 |
|  | *Zoophthora* | 3.857 | 0.0495 |
|  | *Lysobacter* | 3.970 | 0.0463 |
| **Bacterial** | *Bacillus* | 3.857 | 0.0495 |
|  | Unidentified *Planococcaceae* | 3.857 | 0.0495 |
|  | *Streptococcus* | 3.857 | 0.0495 |
|  | Unidentified *Methylobacteriaceae* | 3.857 | 0.0495 |
|  | Unidentified *Burkholderiales* | 3.857 | 0.049534613 |
|  | Unidentified *Comamonadaceae* | 3.857 | 0.0495 |
