# Supplementary material for: Apple endophytic microbiota of different rootstock/scion combinations suggests a genotype-specific influence
Source: Microbiome. 2018 Jan 27;6:18. doi: 10.1186/s40168-018-0403-x (PMC5787276; doi:10.1186/s40168-018-0403-x)
Supplement: Supplementary file 4 — P values of the comparisons between scions regardless of the rootstock they were grafted on using Alpha (observed OTUs and Shannon index) and beta diversity based on Bray Curtis metric. (DOCX 18 kb) [file 40168_2018_403_MOESM4_ESM.docx]

**Table S3.** *P*-values of the comparisons between scions regardless of the rootstock they were grafted on using Alpha (observed OTUs and Shannon index) and Beta diversity based on Bray Curtis metric.

|  | **Comparison** | **Beta diversity (Bray Curtis)** | **Alpha**  **(ObservedOTUs)** | **Alpha**  **(Shannonindex)** |
| --- | --- | --- | --- | --- |
| **Fungal** | ‘Royal Gala’ vs. ‘Honey Crisp’ (regardless of RS) | 0.003 | 0.018 | 0.003 |
|  | ‘Golden Delicious’ vs. ‘Honey Crisp’ (regardless of RS) | 0.003 | 0.002 | 0.004 |
|  | ‘Royal Gala’ vs. ‘Golden Delicious’ (regardless of RS) | 0.056 | 0.038 | 0.065 |
|  | ‘Golden Delicious’/‘M9’ vs. ‘Golden Delicious’/‘M11’ | 1.0 | 0.709 | 0.085 |
|  | ‘Honey Crisp’/‘M9’ vs. ‘Honey Crisp’/‘M111’ | 0.364 | 0.352 | 0.19 |
|  | ‘Royal Gala’/‘M9’ vs. ‘Royal Gala’/‘M111’ | 0.591 | 0.528 | 0.097 |
| **Bacterial** | ‘Royal Gala’ vs. ‘Honey Crisp’ (regardless of RS) | 0.062 | 0.028 | 0.047 |
|  | ‘Golden Delicious’ vs. ‘Honey Crisp’ (regardless of RS) | 0.32 | 0.259 | 0.276 |
|  | ‘Royal Gala’ vs. ‘Golden Delicious’ (regardless of RS) | 0.57 | 0.462 | 0.504 |
|  | ‘Golden Delicious’/‘M9’ vs. ‘Golden Delicious’/‘M11’ | 0.208 | 0.717 | 0.671 |
|  | ‘Honey Crisp’/‘M9’ vs. ‘Honey Crisp’/‘M111’ | 0.102 | 0.108 | 0.098 |
|  | ‘Royal Gala’/‘M9’ vs. ‘Royal Gala’/‘M111’ | 0.092 | 0.19 | 0.435 |
