# Supplementary material for: Apple endophytic microbiota of different rootstock/scion combinations suggests a genotype-specific influence
Source: Microbiome. 2018 Jan 27;6:18. doi: 10.1186/s40168-018-0403-x (PMC5787276; doi:10.1186/s40168-018-0403-x)
Supplement: Supplementary file 5 — ANOVA analysis of the PCoA showing the impact of the grafting on the apple cultivars. (DOCX 17 kb) [file 40168_2018_403_MOESM5_ESM.docx]

**Table S4.** ANOVA analysis on the PCoA showing the impact of the grafting on the apple cultivars.

|  |  | ‘Royal_Gala’_‘M.9’ | Ungrafted_  ‘M.9’ | ‘Golden_Del.’  _‘M.9’ | ‘Honey Crisp’_  ‘M.9’ | ‘Royal_Gala’ _‘M.M.111’ | Ungrafted_  ‘M.M.111’ | ‘Golden_Del.’_  ‘M.M.111’ |
| --- | --- | --- | --- | --- | --- | --- | --- | --- |
| Fungi | Ungrafted_‘M.9’ | 0.09753 |  |  |  |  |  |  |
|  | ‘Golden_Del.’ _‘M.9’ | 1 | 0.0551 |  |  |  |  |  |
|  | ‘Honey Crisp’_ ‘M.9’ | 0.0002088 | 0.01677 | 0.0001923 |  |  |  |  |
|  | ‘Royal_Gala’ _‘M.M.111’ | 0.9951 | 0.313 | 0.9623 | 0.0003322 |  |  |  |
|  | Ungrafted_ ‘M.M.111’ | 0.0003909 | 0.08871 | 0.0002952 | 0.9834 | 0.00105 |  |  |
|  | ‘Golden_Del’._ ‘M.M.111’ | 0.8863 | 0.008341 | 0.9701 | 0.0001766 | 0.5011 | 0.0001889 |  |
|  | ‘Honey Crisp’_ ‘M.M.111’ | 0.0002139 | 0.01903 | 0.000195 | 1 | 0.0003528 | 0.9893 | 0.0001769 |
|  |  | Ungrafted_ ‘M.9’ | ‘Golden_Del.’_‘M.9’ | ‘Honey Crisp’ _ ‘M.9’ | ‘Royal_Gala’_ ‘M.M.111’ | Ungrafted_ ‘M.M.111’ | ‘Golden_Del.’_ ‘M.M.111’ | ‘Honey Crisp’_ ‘M.M.111’ |
| Bacterial | Ungrafted_‘M.9’ |  | 1 | 0.5726 | 0.6204 | 0.1294 | 0.6223 | 0.9999 |
|  | ‘Golden_Del.’ ‘M.9’ | 0.1487 |  | 0.634 | 0.6814 | 0.1537 | 0.6833 | 0.9994 |
|  | ‘Honey Crisp’_ ‘M.9’ | 2.535 | 2.387 |  | 1 | 0.9266 | 1 | 0.3964 |
|  | ‘Royal_Gala’ _’MM.111’ | 2.42 | 2.271 | 0.1156 |  | 0.9003 | 1 | 0.4388 |
|  | Ungrafted_  ‘M.M.111’ | 4.051 | 3.902 | 1.515 | 1.631 |  | 0.8991 | 0.07516 |
|  | ‘Golden_Del.’_ ‘M.M.111’ | 2.415 | 2.266 | 0.1203 | 0.004745 | 1.636 |  | 0.4406 |
|  | ‘Honey Crisp’_ ‘M.M.111’ | 0.4521 | 0.6008 | 2.987 | 2.872 | 4.503 | 2.867 |  |
