# Supplementary material for: Apple endophytic microbiota of different rootstock/scion combinations suggests a genotype-specific influence
Source: Microbiome. 2018 Jan 27;6:18. doi: 10.1186/s40168-018-0403-x (PMC5787276; doi:10.1186/s40168-018-0403-x)
Supplement: Supplementary file 6 — The fungal and bacterial genera that were significantly present at different abundance, according to Kruskal-Wallis comparisons. Description - Table S5. The fungal and bacterial genera that were significantly present at different abundance between “Golden Delicious” when grafted on M.M.111 and M.9. Table S6. The fungal and bacterial genera that were significantly present at different abundance between “Royal Gala” when grafted on M.M.111 and M.9. Table S7. The fungal and bacterial genera that were significantly present at different abundance between “Honey Crisp” when grafted on M.M.111 and M.9. (DOCX 18 kb) [file 40168_2018_403_MOESM6_ESM.docx]

**Table S5.** The fungal and bacterial genera that were significantly present at different abundance between ‘Golden Delicious’ when grafted on MM.111 and M.9, according to Kruskal Wallis comparisons.

|  | **OTU** | **Test-Statistic** | ***P*-value** |
| --- | --- | --- | --- |
| **Fungal** | unidentified *Pezizaceae* | 4.35483871 | 0.036903954 |
|  | unidentified *Ustilaginaceae* | 4.35483871 | 0.036903954 |
|  | *Entoloma* | 3.857142857 | 0.049534613 |
|  | *Piloderma* | 3.857142857 | 0.049534613 |
|  | Unidentified Fungi | 3.857142857 | 0.049534613 |
| **Bacterial** | *Veillonella* | 3.9705882353 | 0.0463015949 |
|  | *Legionella* | 3.9705882353 | 0.0463015949 |
|  | unidentified *Bradyrhizobiaceae* | 3.8571428571 | 0.0495346134 |
|  | *Sphingopyxis* | 3.8571428571 | 0.0495346134 |
|  | unidentified *Comamonadaceae* | 3.8571428571 | 0.0495346134 |
|  | *Pseudomonas* | 3.8571428571 | 0.0495346134 |
|  | unidentified *Xanthomonadaceae* | 3.8571428571 | 0.0495346134 |

**Table S6.** The fungal and bacterial genera that were significantly present at different abundance between ‘Royal Gala’ when grafted on MM.111 and M.9, according to Kruskal Wallis comparisons.

|  | **OTU** | **Test-Statistic** | ***P*-value** |
| --- | --- | --- | --- |
| **Fungal** | *Hypocreaceae unidentified* | 4.35483871 | 0.036903954 |
|  | *Acremonium* | 4.35483871 | 0.036903954 |
| **Bacteria** | *Veillonella* | 4.3548387097 | 0.0369039536 |
|  | *Cellulosimicrobium* | 3.9705882353 | 0.0463015949 |
|  | *Brevibacillus* | 3.9705882353 | 0.0463015949 |
|  | *Sphingomonas* | 3.9705882353 | 0.0463015949 |
|  | *Paenibacillus* | 3.8571428571 | 0.0495346134 |
|  | *Streptococcus* | 3.8571428571 | 0.0495346134 |
|  | *Sphingopyxis* | 3.8571428571 | 0.0495346134 |
|  | *Comamonadaceae* | 3.8571428571 | 0.0495346134 |
|  | *Methyloversatilis* | 3.8571428571 | 0.0495346134 |
|  | *Xanthomonadaceae* | 3.8571428571 | 0.0495346134 |

**Table S7.** The fungal and bacterial genera that were significantly present at different abundance between ‘Honey Crisp’ when grafted on MM.111 and M.9, according to Kruskal Wallis comparisons.

|  | **OTU** | **Test-Statistic** | ***P*-value** |
| --- | --- | --- | --- |
| **Fungal** | *Genea* | 4.35483871 | 0.036903954 |
|  | *Metschnikowia* | 4.35483871 | 0.036903954 |
|  | *Synchytrium* | 3.970588235 | 0.046301595 |
|  | *Cladosporium* | 3.857142857 | 0.049534613 |
|  | *Pyronemataceae*unidentified | 3.857142857 | 0.049534613 |
|  | *Jattaea* | 3.857142857 | 0.049534613 |
|  | *Tylopilus* | 3.857142857 | 0.049534613 |
| **Bacterial** | *Actinomyces* | 4.3548387097 | 0.0369039536 |
|  | *Lactococcus* | 4.3548387097 | 0.0369039536 |
|  | *Fusobacterium* | 4.3548387097 | 0.0369039536 |
|  | *Methylobacteriaceae* | 4.3548387097 | 0.0369039536 |
|  | *Novosphingobium* | 4.3548387097 | 0.0369039536 |
|  | *Betaproteobacteria* | 4.3548387097 | 0.0369039536 |
|  | *Anaerococcus* | 3.9705882353 | 0.0463015949 |
|  | *Planococcaceae* | 3.8571428571 | 0.0495346134 |
|  | *Streptococcus* | 3.8571428571 | 0.0495346134 |
|  | *Peptoniphilus* | 3.8571428571 | 0.0495346134 |
|  | *Comamonadaceae* | 3.8571428571 | 0.0495346134 |
|  | *Methyloversatilis* | 3.8571428571 | 0.0495346134 |
|  | *Lysobacter* | 3.8571428571 | 0.0495346134 |
